# Extracts

**Published:** 1882-01

**Authors:** 


### Extracts.

VACCINATION WITH CALF LYMPH.—It may be safely asserted that the use of virus direct from the animal ensures safty from scrofula, syphilis, cutaneous diseases, pus inoculation aud more especially imperfect vaccination from the use of deteriorated lymph.

Vaccine virus, indigenous to the heifer, does not degenerate by frequent transmission through the animal, but, when removed to a foreign soil—the human subject—it undergoes modification, and if the greatest care is not observed, is liable to undergo very serious degeneration, for it cannot be doubted that a very gradual but imperceptible change does take place from one transmission to another, sometimes more perceptible in one case than another.

One of the reasons why old practitioners preferred a crust for vaccination from. was because crusts of a typical character never form where the vesicles have been imperfect either in type or development, and hence by a continual survival and reproduction of the fittest they were able to go on for years without much apparent degeneration of the lymph in use.

Perfect vaccinia is always attended with profound constitutional fever, and this is much more marked where heifer lymph is used than where exhausted virus of long human transmission has been employed, and is usually coincident with the rise, development and decline of the areola which begins on the middle of 8th and lasts until 12th day.

In my own judgment a better test of a perfect vaccination is the production of characteristic vesicles, passing through all their several stages of development, decline and fall of crust, leaving behind them indellible cicatrices or depressed scars of the peculiar and well-defined type.

The resulting vaccine scar is a matter of great importance, and offers to the observing practitioner an excellent guide with respect to the perfection of a vaccination, owing to the direct relation between the two. A great variety of vaccine scars are to be met with, while there is but one typical of a perfect vaccination.

These variations from the normal type may be accounted for in the following manner:

They may result: 1st. From the use of lymph enfeebled by a long series of human transmissions. 2d. Some imperfect condition of the virus—however pure—preventing its proper development, or an unsusceptibility on the part of the patient; or 3d. Violence applied to the vesicle by which it is lacerated, as from scratches, adherent clothing, etc.

Ninety per cent. of the variations are due to the first cause, a smaller number to the second, and fewer still to the third. Since in all but the very feeble good virus will produce a perfect vesicle followed by a typical scar, even rupture of the vesicle, while modifying, fails to prevent the formation of a characteristic cicatrix.

Dr. Willan describes three typical spurious vaccinal results:

1st. A single pearl-colored vesicle, less than the genuine. The top flattened, but the margins not rounded nor prominent. It is set on a hard base, slightly elevated, with an areola of a dark rose color.

2d. This is cellular, like the genuine vesicle, but smaller, and with a sharp angulated edge, areola pale red and very extensive.

3d. A vesicle without an areola.

None of these afford any security against small-pox.

Three causes are suggested to account for the failures:

1st. From matter having been taken from a spurious vesicle, or from a genuine vesicle at too late a period.

2d. From a patient being seized soon after vaccination with some contagious fever, as measles, scarlatina, etc.

3d. From the patient having been affected at the time of inoculation with some chronic skin disease, as tinea, lepra, etc. These eruptions always disappear after vaccination. And,

4th. It has been supposed that in arm-to-arm vaccination puncturing the vesicle in order to take lymph from it, by disturbing the process of development, may destroy its prophylactic influence.

The advantages to be derived from the use of heifer-transmitted lymph may be briefiy enumerated as follows:

1st. Animal vaccine guarantees against the possibility of transmitting any diseased contamination. Cutaneous disease, syphilitic or septic contamination are quite unknown as a sequence.

2d. It enables a constant supply of pure lymph to be kept up, on which to draw in an emergency.

3d. It gives the greatest possible guarantee of protection by enabling the practitioner to carry out true Jennerian vaccination, which has been amply proven by the recorded experiments of Woodville and others to be permanently protective.

4th. It enables vaccination to be carried on without reference to those already vaccinated or the necessity of our tapping a vesicle to obtain humanized lymph, thereby rendering it less protective, and it prevents a vaccine famine.

It has been objected to vaccination with animal lymph that,

1st. It is too violent.

2d. It has been asserted that animal vaccine might communicate diseases from the animal.

3d. That it is difficult to *take* unless used when quite fresh.

This is the only objection worth considering, which may be met and overcome by trying to understand and appreciate the fact that bovine albumen is much less soluble than the human, and therefore special care is necessary to liquify the lymph on the ivory point by dipping in cold water previous to using, and rubbing firmly over the scratches to remove the lymph effectually. This quite overcomes the difficulty.—*W. E. Bessey, M.D., in Canada Med. Record.*

---

Adjuvants, Corrigents, etc.—Opium and veratria appear to have a degree of antagonism when used in toxic doses, but I am aware of no published observations upon their effects when used therapeutically.

Conium and hyoscyamus act together somewhat similarly to opium and conium.

A combination of opium and hyoscyamus, according to Harley, forms one of the most powerful of narcotic combinations.

Conium with belladonna behaves similarly to conium and hyoscyaums.

Caustic potash and soda decompose atropia out of the body, but, given internally, appear to have no effect on the action of belladonna. Caustic ammonia and lime-water should never be added to mixtures containing belladonna, as they completely neutralize its effects when they have remained two or three days in contact.

Digitalis often fails to act as a diuretic until combined with bicarbonate of ammonia or with squill.

In speaking of the diuretic effect of digitalis, it may be remarked that there is some ground for a belief that the diuretic principle of the drug is not soluble in alcohol, and that digitoxin, which appears to influence the action of the heart most powerfully, is not very soluble in water. Therefore, in using digitalis therapeutically, the infusion should not be chosen as a cardiac remedy, nor the alcoholic preparation when diuretic action is desired.

It may here be observed that infusion of digitalis, if made as other infusions are not unfrequently prepared, from the fluid extract, will, as a rule, be found devoid of diuretic effect.

Iron may be given with potash when the latter is used in the alkaline treatment of gout, and by its effect in maintaining the composition of the blood avoids the destructive effects of the potassium salt upon this fluid. It has a similar effect when given with mercury, as when the latter is taken for long periods and large doses in cases of syphilis.

When camphor is administered with cantharides, the effects of the latter upon the kidneys and bladder will be more or less obviated. As camphor does not pass through the kidneys, it is possible that it forms some combination with the cantharidin which prevents its escape through that channel.

Strychnia has been found to serve as a respiratory stimulant in dyspnœa caused by opium.

Chloral has been found to be an antidote to the toxic effects of strychnia.

Ammonia, occasionally administered by inhalation during chloroform-anæsthesia, prevents heart-failure.

Another instance of the value of combination, is the recent demonstration of the value of sulphuric ether as a corrigent for cod-liver oil when internally administered.

Carbonate or spirit of ammonia, given with iodide of potassium, are said to control the symptoms of iodism in some cases.

Opium renders iodide of potassium inert.—*F. A. Castle, M.D., in N. Y. Med. Record.*

Occlusion of the Gravid Uterus.—As soon as the diagnosis is declared, the proper treatment must certainly be an early recourse to incision: that is, after the failure of endeavors to open the os by the finger, probe, or sound, and after sufficient time has been allowed to determine whether the uterine contractions could overcome the occlusion. Further delay could not be otherwise than disastrous to the mother or child, or to both, as it has been in several cases recorded by Ashwell and other authors.

Craniotomy and the forceps have been required, after incisions, to accomplish the delivery; these procedures were doubtless necessary, owing to the long delay before recourse was had to incision, or, possibly, may have been required, owing to some peculiarity of the case, independent of the occlusion.

Incision, crucial or multiple, is the treatment for occlusion, which should be resorted to as soon as certainly detected. I would advise, both for the purpose of discovering and diagnosticating this rare but troublesome lesion, and for the more intelligent and efficient performance of the incision, that a speculum be employed.—*Joseph A. Eve, M.D., in Gynecological Trans.*, 1881.

---

Treatment of Leucorrhea in Children.—Leucorrhea in children, says M. Bouchut, (*Practitioner; from Le Practicien*) is caused by vulvitis, not vaginitis or metritis. He treats this condition by extreme cleanliness, repeated bathing with bran-water and lead-water, lotions of corrosive sublimate (two grains to ten ounces of water), carbolic acid (two grains to the ounce), and occasionally solution of nitrate of silver (three grains to the ounce). In the intervals of applying the lotions a pledget of lint saturated with coal-tar or an ointment of red precipitate may be placed between the labia. Such a pledget kept in place by a pad protects the surrounding parts as well as the labia themselves from the irritating secretion, which is often present in considerable quantities. For the general treatment, M. Bouchut recommends the administration of cod-liver oil and quinine to strumous patients, and of arsenic to those with eczematous eruptions.—*Ohio Med. Journal.*

Leucorrhea in children is often, if not generally occasioned by the migration of seat-worms—ascaris vermicularis—from the rectum into the vagina, and is best treated by the administration of calomel and santonin to remove the cause, and by bathing in salt-water, observing, of course, thorough cleanliness, and employing such tonics and hygienic management as be indicated.

---

MANAGEMENT OF THE SHOULDERS IN LABOR.—I have never met with a case of ruptured perineum in my own practice, which embraces two thousand midwifery cases. I do not know whether this is owing to good fortune or to the means which I invariably adopt in all cases which I am called on to attend. Of course I have met with slight lacerations of the fourchette, but not of sufficient seriousness to require surgical interference.

The proper plan is, before the head actually commences to impinge on the soft parts, to pass the finger round the whole surface of the perineum, inside, during the pain, and attenuate the tissues by drawing them downwards and backwards. This kind of *massage*, so to speak, is of great service in preparing the perineum for the severe strain it is about to undergo. When the pains are of a violently forcible character it is necessary, of course, to guide the head and control its movements; but if the soft parts be properly prepared in the manner I have suggested, the perineum may be readily slipped under the chin, and the term of the labor thereby greatly shortened. I might now suggest in addition, the proper management of the glottis and extension of the left leg at this stage to produce relaxation of the sphincters. The abduction and flexion of the limbs are proper until the soft parts are completely stretched; then the extension of the left leg adds to the safety of the perineum by its relaxation and the increase in the degree of its inclination. These remarks apply more particularly to the management of the head, but they also have a bearing, as you will see hereafter, on the delivery of the shoulders. A great rest usually takes place after the delivery of the head, particularly in primiparæ. The young obstetrician at this stage awaits anxiously for a renewal of the pains and sees with horror the face of the child becoming livid. Fearful for its safety, he immediately commences to pull on the head forcibly downwards and backwards. A sudden and violent pain is excited by his efforts; the sphincter contracts and the shoulders are suddenly expelled, tearing the perineum in their rapid course.

The better plan, however, and the one I always adopted in cases of primiparæ, is to deliver each shoulder separately. After the proper rotation of the shoulders, which should be done very gently, I pass two fingers up into the axilla of the arm presenting at the pubis, gently depressing the head in this movement. I then raise the head up towards abdomen of the mother, and in like manner deliver the remaining shoulder. The first shoulder should, if possible, be delivered before the pains re-commence, after the delivery of the head. If I do not succeed with two fingers I do not hesitate to pass the whole hand and draw down the arm. This is sometimes a little painful to the mother, but it invariably saves the perineum. The great frequency of rupture of the perineum by the shoulders is due to the fact that they are too often disregarded in the management of the labor. The head being delivered without injury to the soft parts, the accoucheur thinks all difficulty is over; but this is a very great error. The shoulders form abrupt stumpy projections which are very apt to cut the attenuated parts if not properly watched and controlled.

I have spoken of the proper management of the glottis as a means of saving the perineum.

The more outcry the woman makes at the terminal stage of labor—that is when the head and shoulders are about to pass—the better.

The woman, therefore, should be encouraged to cry out at this crisis. Her very distress seems to be the means devised to save her from future injury.

Unfortunately, in our times, it seems that more pains are taken to look for injuries to the perineum than to guard against them. The whole system of midwifery formerly taught in the schools has been reversed by modern practice. The gynecologist appears to have the place in a great measure of the obstetrician. Women are now turned up and examined immediately after delivery in the search for lesions of the genitalia. I was greatly surprised at a meeting of the Obstetricial Section of the Medical and Chirurgical Faculty, last week, to discover that this practice is the unvarying rule of every member who was present. The old masters of midwifery would have looked with horror upon procedures of this character, and I beg leave as one of their pupils to protest earnestly against this unnecessary, if not indelicate, innovation.—*John Morris, M.D, in Maryland Med. Journal.*

---

Removal of Foreign Bodies from the Ear.—A foreign body in the auditory canal, unlike a foreign body on the cornea or in the eye, is a very harmless thing if properly treated. Even delay is not dangerous. It is true that a foreign body which has passed the auditory canal and entered the tympanic cavity is dangerous; but even then its danger, as regards life, is infinitesimal, if only it be left alone. The worst consequences usually are loss of the middle portion of the ear, and consequently of all acute hearing power.

As I said a few moments ago, the alarm which is caused in the minds of the friends is the most striking symptom of the presence of a foreign body in the auditory canal. Let me say to you, then, at the very outset, do not be excited when a patient is brought to you who is said to have a foreign body in the ear. Let me tell you, in the second place, never to believe that there is a foreign body in the auditory canal until you have seen it with your eyes, if you are the person who is to remove it. Take no man's, no woman's, no nurse's, take no person's word for this. Very bad results have occurred from the neglect of what seems to be a fundamental proposition. Ears have been syringed—much worse, have been treated with instruments; membrana tympani have been broken, ossicles have been dragged out, labyrinths have been invaded, and meningitis and death have been caused, by neglect of this simple rule. In other words, attempts have been made to remove foreign bodies from the auditory canal when there were none there. When you have time to review the literature of this subject you will find it sensational reading, and such as reflects no credit upon medical practice.

How shall we examine the auditory canal supposed to contain a foreign body?

In lieu of the mirror, which may not be at hand in some instances, a piece of looking glass answers very well; and a speculum can be easily extemporized by means of pasteboard. I speak of these things because I wish to impress upon your minds that he only is a competent surgeon, he only is thoroughly at home in his profession, who can make the simplest appliances do his work. The picture of Alpheus Crosby boring the mastoid process with a gimlet, that of the surgeon amputating a leg with a jack-knife, are always very pleasant ones for the American medical profession to contemplate. They show great capabilities under difficulties.

But let us discuss the simple cases first—those in which the foreign body has been pushed in simply with the child's fingers, or where it has, as in the case of insects, entered by its own volition. What are you to do? Here we enter upon ground, which, until within the last few years, I had supposed was settled.

Nay, more, I had supposed it to be so well established that, in the case of any foreign body in the ear, a thorough attempt, by means of the syringe and warm water, should be made before any measures are employed. But those of us who believe this—those of us who, as we thought, had undone the damage done by the writers of the dark ages who attacked a foreign body as if it were a burglar, and hunted it down as if it were a fugitive from justice, have been confronted by passages in recent books from authorities on aural disease, which claim that those who use the syringe are clumsy and bunglers, and that removal by this instrument is a method never to be taught. As strong language as this has been used. But, after careful consideration of all aspects of the subject, I I am not convinced by these recent writers. On the contrary, I think the teachings of these writers are dangerous, and that the employment of forceps, and ingenious extractors will take us back to the times of meddlesome surgery. There can be nothing more cleanly, nothing more harmless, than a stream of pure warm water, from the mouth of a syringe, directed upon a foreign body, so that the returning wave shall sweep it out; and it only needs to be fairly considered, in contradistinction to beautifully contrived forceps, admirably arranged snares, and all the other armamentarium of those who sneer at the syringe, in order to meet with your warmest approval. If, however, it has been pushed far in, or has lain long enough to swell, it will be sometimes impossible, after the most thorough trial, to remove it with the return wave of water. It will then be necessary to resort to the most careful surgical interference, and this must be done while the patient is under the influence of an anæsthetic, and the parts must be thoroughly illuminated by means of the mirror.

If you chance to be in the woods with a friend into whose ear an insect has entered, the simplest method of removing it is to pour in some water, warmed by the hand, until the insect can be dislodged. When flies and maggots gain entrance to suppurating ears, the case is one for simple treatment with the syringe, first using some agent which will kill the fly or maggot, such as carbolic acid, Labarraque's solution, or the like; after this, the stream of warm water may be employed. An attempt to remove them with the forceps only makes them more lively and increases the difficulty of their removal.

I venture to recapitulate: First, be sure that there is a foreign body in the ear. Second, remove it by syringing, if possible. Third, wait quietly, if it is so wedged in that it cannot be removed in this manner, until you can secure competent assistance, and proceed with care and caution, and with such instruments as your ingenuity and the ordinary instrumental collections of aural surgeons will furnish.—*D. B. St. John Roosa, M.D., in New York Med. Record.*

---

THE TREATMENT OF CONSUMPTION.—The impaired nutrition is not a cause, but an effect, of a vicious transmission of heredity, or the sequel of a broken down pneumonia.

Except as palliatives, we are in dense ignorance of specifics; but we do know—and the sooner the fact is generally admitted, the better will be the statistics of mortality—that the liver is always a keen and sympathetic sufferer, and that to its languidly performed functions may be ascribed the gastric disturbances of these patients. The daily exhibition of cod-liver oil and whisky, of cod-liver emulsions, of pure cod-liver oil, and of the endless variety in the preparation of this unpalatable medicine, is bad practice, since it defeats the very end that the physician has in view from its administration. At the best, cod-liver oil is only a constructive, and not a thoroughly good one at that. While given with the express view of overcoming tissue-waste, it, in fact, increases it by creating a repugnance to food, by exciting nausea, by inducing turbulent action in the stomach, and by preventing the assimilation of itself or anything else, from its own inherent defects. Few patients can tolerate it; many with delicate digestion abhor it. To force it upon such cases is pure empiricism, for no effect can be expected, except the very vague one of construction.

As the disease advances, the gastric difficulty is intensified, and the greatest care and discrimination are demanded from the physician in the exhibition of food and medicine. The distressing cough, the colliquative sweats and diarrhœa, are all alleviated more certainly by proper attention to the nutritive processes, than by pollydosage, addressed simply to each symptom.

Except in the rare instances where it is well-borne, I never use cod-liver oil, but prefer as a constructive some preparation of malt; maltine with peptones is the best I know of, and is generally pleasant to the patient. It is a first-rate heat-producer, a good starch-digestive, and an excellent fat-former. In cases of acute dyspepsia, pepsin and pancreatine may be added, with benefit to the maltine.

Active medication, addressed simply to each symptom, is contra-indicated. What is demanded is an enriched blood-supply, and as gradually this is brought about, and the centres participate in the generally improved nutrition, all of the characteristics of impoverished supply disappear, one by one. I hold it to be proven that most of the distressing coincidences of consumption call for little medication, except that which shall prevent tissue-waste; and the more advanced our studies in this direction, the more satisfactory will our results be.

In closing, I wish to impress my conviction clearly: First, that, in the majority of cases, cod-liver oil is not only inert, but absolutely injurious; second, that much medicine is not demanded; third, that the liver and digestive functions call for the most interference; fourth, that we accomplish more by improving nutrition than by any other plan of treatment.—*Horatio R. Bigelow, M.D., in New England Med. Monthly.*

---

Herpes Zoster or Shingles.—After a few days of slight fever, an eruption of small erythematous spots appears along the course of one of the cutaneous nerves. The eruption is generally preceded by a neuralgia, sometimes very severe, for several days. In other cases the pain is entirely wanting. Small papules soon appear in the centre of the spot, which develope into vesicles. These are often umbilicated like small-pox vesicles. You remember that when speaking of the anatomy of vesicles, I said that umbilication was not peculiar to the vesicles or pustules of small-pox, as stated by some writers. In the course of a week the vesicles dry into thin crusts or change into pustules. In two to three weeks the crusts fall off, leaving pigmented spots or superficial scars to mark the site of the eruption.

The prognosis is favorable. There is a very widespread opinion current among the laity that if a belt of "the shingles" crosses the middle line of the body and extends to the other side, or if it is bilateral, the disease will certainly be fatal. This notion, though frequently disproved by cases of bilateral zosters which did not terminate fatally, has persisted for nearly two thousand years in the medical prognostics of the people.

The treatment is extremely simple. We will apply something to protect the vesicles against accidental injury from friction of the clothing or otherwise, and the best thing for that is cotton batting.

The vesicles will be covered by a layer of cotton batting, which will be kept in place by a few turns of a roller bandage. He suffers intensely from the pain, and for that we will give him one-fourth of a grain of sulphate of morphia every four hours, or oftener if necessary. You may apply a dusting powder of precipitated chalk and oxide of zinc, with a little morphia, to satisfy the patient that you are doing something for him.

If the neuralgia persists after the disappearance of the eruption, you may use hypodermic injections of morphia or atropia, or the two combined over the track of the nerve. The constant current may also be used with success in chronic cases.—*George H. Rohe, M.D., in Maryland Medical Journal.*

---

Sulphur for Pimples on the Face.—Dr. Gage Parsons believes that Mr. Erasmus Wilson was the first to propose sulphur lotion in acne punctata, according to the *Practitioner.* The usual lotion of the flowers of sulphur with glycerine and water is undoubtedly a valuable remedy, but from the readiness with which the sulphur separates it is inelegant and inconvenient, while it is not quite satisfactory in its results. A far more efficacious mode of using sulphur is to dust the face with pure precipitated sulphur every night with an ordinary puff used for toilet purposes. Recently two severe cases of acne of two years' standing, which had resisted the ordinary methods of treatment, yielded at once to sulphur thus applied. If the sulphur be scented with oil of lemon or roses it will form an elegant cosmetic.—*Canada Med. Record.*

---

Popularizing Medicine.—The *Family Doctor* is the name of a new publication which, though "edited by a physician," it is difficult to regard as anything else than a journalistic error. The plan of popularizing medicine is a serious mistake, and any medical information, of a desirable or necessary kind for general use, can only rightly be such as to find admission to the pages of hygienic journals and health reviews.—*Med. Press and Circular.*

